# Impact of a local, coastal community based management regime when defining marine protected areas: Empirical results from a study in Okinawa, Japan

**DOI:** 10.1371/journal.pone.0213354

**Published:** 2019-03-08

**Authors:** Payal Shah, Sahan T. M. Dissanayake, Yoko Fujita, Paulo A. L. D. Nunes

**Affiliations:** 1 Okinawa Institute of Science and Technology Graduate University, Onna-son, Okinawa, Japan; 2 Portland State University, Portland, Oregon and IUCN-Sri Lanka, Colombo Sri Lanka; 3 University of the Ryukyus, Okinawa, Japan; 4 Climate, Biodiversity, Land and Water Department, Food and Agriculture Organization of the United Nations, Rome, Italy; Shandong University of Science and Technology, CHINA

## Abstract

There is a growing impetus to increase marine protected areas coverage globally from 6% to 30% in 2030. Successfully establishing and maintaining marine protected areas require incorporating public preferences into their establishment and management. We investigate the role of alternate management regimes (top-down and bottom-up) on preferences for marine protected areas and the marginal rate of substitution between natural and man-made capital using a case study in the Asia-Pacific region of Okinawa, Japan. We implemented a choice experiment survey to infer which attributes of marine protected areas are most important for the respondents. We use our survey results to calculate respondents’ willingness to support marine protected areas in Okinawa. This study contributes to the policy debate on management of marine protected areas with empirical data that characterizes the perception of Okinawan residents with respect to the role of local coastal communities (bottom-up) compared to central government based agencies (top-down) management. We extend the analysis and estimate the trade-offs to residents in Okinawa between natural capital (i.e. coral coverage and marine biodiversity attribute) and man-made capital (i.e. restrictions on coastal development). We find that the underlying management regime affects the local residents’ valuation of the marine protected area with residents showing a higher willingness to support bottom-up management regimes. There is also substantial differences in the willingness to support different characteristics of marine protected areas by management type. Finally, we find that the marginal rate of substitution between natural capital and man-made capital varies by management type such that residents would need to be compensated relatively less in terms of man-made capital in the presence of a policy scenario that proposes an increase in natural capital under a bottom-up management regime.

## Introduction

Today marine protected areas (MPAs) cover almost 6% of the earth’s coastal and marine areas. The International Union for Conservation of Nature (IUCN) has adopted scientists’ recommendation that world leaders should conserve at least 30% of the ocean by 2030 to maintain biodiversity, boost fisheries productivity, and safeguard the myriad economic, cultural, and life-supporting benefits of the sea. Chile and the Arctic are two examples that are already moving in this direction. Recently, Chile established the world’s largest MPA, Rapa Nui National Park, roughly the size of Chile’s land area, aiming to safeguard the waters surrounding the Pacific Ocean islands. Another large marine area covering 1.5 million square kilometers, an area about five times the size of Germany, in the Weddell Sea and around the Antarctic Peninsula, has been agreed upon to be set aside for protection that will ban fishing and safeguard species such as penguins, killer whales, leopard seals and blue whales. In this context, Japan, the focus of this study, is also increasing its share of coastal and marine areas dedicated to conservation, especially for the conservation of reef-building corals and the associated marine biodiversity mainly found in its southernmost prefecture of Okinawa. In 2014 the Ministry of Environment of Japan designated the Kerama Islands in Okinawa as the country's 31st national park, protecting approximately 1000 square kilometers of marine and surrounding terrestrial areas.

The push to increase MPA coverage globally and in Japan has led to a growing interest in issues of MPA management. Recent studies argue that MPA design and management are key determining factors in their success or failure [1]. In [2], the author uses case studies of Indonesia, Philippines, Fiji and Okinawa to argue about the advantages and disadvantages of different MPA management regimes. Several other studies have also highlighted the role of institutional background and management schemes for MPAs in Japan [3–5]. This is particularly important because MPA design and management can often focus largely on ecological outcomes and ignore the potential adverse impact that MPA establishment has on the socio-economic aspects of local communities. However, studies have shown that the success of MPAs depends on the support from local people and communities [6]. Such local support depends on a range of issues such as the perception of the local people on the potential socio-economic and ecological outcomes, the effectiveness of management policies, gender-based differences in the level of support, etc. [7–10]. Previous studies find that conservation policies that reconcile the differences between ecological criteria for MPA creation with socio-economic concerns of the different stakeholders involved are relatively more successful [11–12]. In [13], the authors identify the most important factors that drive the success and failure of MPAs. Their key finding, based on 27 MPA case studies across the world, is that stakeholder engagement is the most important factor affecting a MPA’s success and the absence of stakeholder engagement is the most important factor influencing a MPA’s failure.

We conduct an economic valuation study in Okinawa to investigate the impact of management aspects on the local support for MPAs in the area. We use a choice experiment (CE) survey, a non-market valuation method, to assess the preferences and values related to the provision of public goods, such as MPAs. Previous studies have used CE methods and other stated preference methods to quantify the value of marine environments [14–24]. In [16], the authors use CE methods to estimate the value of extractive and non-extractive uses of Guam’s coral reefs. In [17] and [19], the authors use CE methods to evaluate preferences for different management options for deep-sea coral conservation in Ireland and find large public support for marine protection. In [20], the authors use a combination of economic valuation tools (including CE methods) to identify spatial variation in the commercial and recreational value of coral reefs across Bonaire. In [23], the authors use CE methods for evaluating the economic cost of climate change and ocean acidification to scuba diving activity in the MPAs of Medes Islands, Spain.

We focus our study on Okinawa because it is an area of high biodiversity and ecological importance that is threatened by climate change, over-exploitation from anthropogenic factors and increase in point and non-point sources of pollution [25]. Thus, the Ministry of Environment in Tokyo and the local government office (e.g. Nature Conservation Division of Okinawa Prefectural Government) and fishing cooperatives in Okinawa (e.g. Onna Fishery Association) are pushing for new MPA coverage in the region. To provide guidance on local preferences for MPAs, we collect empirical data on Okinawan residents with respect to the role of local coastal communities (also known as “bottom-up” management approach)–compared to central government-based agencies (also known as “top-down” management approach)–on the management of MPAs and the fulfillment of its proposed objectives.

Our study is the first to elicit responses for two different management policies that are prevalent when designing and managing MPAs and also the first to use CE methods for valuation of non-market benefits associated with the marine environment of Okinawa. In previous work, we use the data collected from this CE survey to evaluate how the value of and preferences for key characteristics provided by the marine environment of Okinawa vary among different stakeholders, i.e. residents and tourists, and for different management approaches using separate regression analysis for each sample [26]. We also investigate how perceptions of change in coral reef ecosystems (shifting baselines) affect stakeholders' WTP for the establishment of protected areas [27].

In this paper, we first analyze how the preferences and the WTP vary between a national based MPA management regime and a community based MPA management regime by analyzing the pooled data from the split sample study using management interactions. The national based management regime endorses a top-down approach that is characterized by centrally managed marine areas with strict restrictions on marine activities. Conversely, the community based MPA management regime endorses a bottom-up approach that engages key stakeholders in the decision-making process and is established and monitored by local organizations.

We also contribute to the literature by evaluating the marginal rate of substitution (MRS) between natural capital and man-made capital and how this MRS is impacted by nationally based (top-down) versus community based (bottom-up) MPA management regimes. Recent studies highlight the importance of both natural capital and human or man-made capital in the provision and sustainable use of natural resources [28]. Thus, in addition to understanding the monetary value of the different types of capital associated with environmental goods and services, it is also important to understand the trade-off relationships that exist among them to the beneficiaries of these services. A key concern in cost-benefit analysis of environmental policies including designation of MPAs is the degree of substitutability of environmental goods and services with man-made goods and services. Previous studies find evidence that management practices may be affected by the trade-offs associated between environmental goods and man-made goods [29–30]. We use the valuation results to estimate the MRS between natural capital (i.e. coral coverage attribute) and man-made capital (i.e. restrictions on coastal development) across the two proposed MPA management regimes.

We find there is substantial difference in the preferences for and valuation of MPAs in Okinawa depending on the management regime under consideration. All attributes associated with an MPA are valued highly when the underlying management regime assigns the responsibility of the MPA to a local, coastal community versus a central, Tokyo based government agency. Furthermore, empirical results inform that marginal impacts are not the same across the different attributes under consideration. We also find that the residents of Okinawa place a relatively higher value on natural capital such as coral coverage and marine biodiversity compared to man-made capital such as more infrastructure development in coastal areas. Finally, when analyzing the MRS, we find that residents would need to be compensated relatively less in terms of man-made capital in the presence of a policy scenario that proposes an increase in natural capital under a bottom-up management regime.

## Study area and methods

### Study area

Okinawa is the southernmost prefecture of Japan comprising many small islands covering a terrestrial area of approximately 1200 square km. Okinawa is situated in a subtropical climate and is home to ecologically significant coral reefs situated at the northernmost end of the border between the Pacific and the Indian Oceans. These corals lie within a biodiversity hotspot that supports a high diversity of endemic species, plants and animals [31]. More than 250 species of corals, 360 species of fish, 1640 species of invertebrates and 220 species of seaweed are found in the marine area surrounding Okinawa [32]. Okinawan waters also provide an important habitat for green turtles, loggerheads, and hawksbill turtles, humpback whales and the near extinct dugong [32]http://www.jcrs.jp/old/english/publications/coralreefsofjapan_top.htm. According to [33], the oceans surrounding Okinawa rank high in global marine conservation priority owing to its high level of multi-taxon endemism. The coral reefs are also economically valuable and provide a range of ecosystem goods and services for human consumption, such as recreational opportunities, coastal protection, habitat for fish and other marine species. It is estimated that coral reefs in Japan, mainly found in and around Okinawa, generate as much as $1665 million per year [34].

Okinawa’s marine ecosystem is increasingly threatened by climate change and increased anthropogenic pressures. In [25], the author highlights the increase in degradation and deterioration of the Okinawan marine environment and its rich coral reef ecosystem. Studies find that the key factors adversely affecting Okinawa’s marine environment are climate change, bleaching events of 1998, red soil run-off and ocean eutrophication, coastal reclamation and development, excessive fishing, predation by crown-of-thorn starfish and tourism [25, 35–37]. These stressors have resulted in a significant decrease and degradation of coral reefs in Okinawa. Coral reefs help sustain marine biodiversity and provide essential habitat for numerous fish and other marine organisms. Thus, widespread and persistent damages to the coral reefs endangers marine biodiversity and existence of numerous dependent marine organisms and pose great challenges for human well-being by affecting the provision of essential ecosystem goods and services. To help protect and restore the health of the coral reefs and the marine environment at large, there is an increasing push to expand the MPA coverage in Okinawa, both through community based and national based management regimes.

According to [3], MPAs in Japan (and in Okinawa) can be divided into six broad categories (similar to IUCN categories), three of which function primarily as top-down or centrally managed MPAs and the other three are akin to bottom-up or community-managed MPAs. The three top-down categories of MPAs are administered by the Ministry of Environment and comprise of marine parks, marine special areas and wildlife special protection areas. The other three categories of MPAs are mainly established for conserving fishery related species or aquatic animals and marine environment; such MPAs are generally designed and managed by the prefectural governments or local fishing communities. In [5], the authors describe another category of MPAs in Japan, autonomous MPAs, which do not fall within a legal MPA framework but are implemented by local communities (e.g. local fishermen, local tourism-based shops etc.) based on issue-specific cases.

The Ministry of Environment, the Okinawan prefectural government and local fishing communities have implemented a variety of top-down and bottom-up MPAs to provide protection to the local marine environment. Okinawa has several nationally managed MPAs such as Kabira Bay and Nagura Bay MPAs in Ishigaki island, the marine special area in Sakiyama Bay (designated as protected in 2010) and the recently established marine national park in the Kerama Islands (designated as protected in 2014). The nationally managed MPAs in Okinawa are largely managed by the Ministry of the Environment of Japan and protected year round. These areas were established with the primary objective of conservation of biodiversity and the natural environment. However, such nationally managed MPAs in Japan are often restrictive for local economics (e.g. fisheries) and ill-suited for social and cultural contexts for communities living in vicinity to the MPAs [2].

There are also community based MPAs such as those in local fishing communities (e.g. Onna village, Haneji-Nakijin, and Yaeyama district) and strict no-take and diving zones established by local dive shops and fisheries cooperative association in the Zamami area in 1999. Many community-managed MPAs in Okinawa are established primarily for protection and enhancement of fishery resources and often target protection of specific species such as shellfish, emperor fish and groupers. Such MPAs are important because they actively involve local participation from fishermen and other stakeholders that are closely associated with the marine environment and hence avoid many of the problems caused by national based MPA management regimes. Community-managed MPAs are better at encouraging local participation and compliance and have longer lasting effects compared to nationally managed MPAs [38]. Community-managed MPAs are also more flexible and often allow for adaptive management depending on the changing scenarios [4]. However, while bottom-up MPAs are ideal for addressing issues facing the immediate communities, they are often too small to achieve larger conservation targets. According to [5], bottom-up or community-based MPA design and management that emphasize consensus-building often set conservation targets that are too low and fail to take more drastic actions. The study also highlights the risk of moral hazard by local participants and that such risk is magnified as more stakeholders become involved in the design and management of the MPAs.

Currently there is a lack of knowledge on how the management regime under consideration, i.e. community based MPA management versus a national based MPA management, impact public preferences and WTP for MPAs. We contribute to this knowledge gap using a CE survey to elicit preferences for establishing two types of MPAs in Okinawa: 1) community-based (bottom-up) MPAs that are identified, implemented and managed by local organizations and 2) nationally managed (top-down) MPAs that are identified, implemented and managed by the central government.

### Choice experiment method and survey design

The CE method belongs to the family of stated preferences methods [39–41] but it has the added advantage that the good or policy being evaluated is divided into its key components or attributes. This improves its usefulness in a management context as researchers can elicit preferences for individual attributes and also analyze trade-offs. Participants are asked to make a choice between alternatives with different attribute-levels. A common attribute among most CE studies is price or a proxy variable for price which enables estimating the WTP for each individual respondent. The CE method allows us to infer which attributes are most important for people’s choices, estimate WTP for changes in attributes, and predict WTP for future scenarios with different bundles of attributes [42].

The main component of CE surveys is the choice card, which presents alternate bundles based on different combinations of levels of key attributes/benefits associated with marine protection. The respondents are asked to choose among these alternate bundles. Respondents are typically presented with multiple choice scorecards and are asked to repeatedly choose the best bundle for each scorecard. The first task in designing a CE survey is identifying the relevant attributes and the levels (or values) of that attribute that accurately reflect the good or policy being valued. For this study we first discussed the overall status and primary reasons for establishing MPAs in Okinawa with members from the Nature Conservation section and the Fisheries Extension center from the Okinawan prefectural government offices. We used this information along with literature on the concerns and pressures facing Okinawa’s marine environment to design preliminary survey instruments. We then conducted two focus group studies in February 2014. The first focus group was conducted among 12 faculty, staff and students from the University of Ryukyus. The second focus group was conducted two weeks after the first focus group study among a group of 12 residents of Onna village in Okinawa. The results from the focus group studies helped us revise the language and certain key aspects of the survey. We then conducted a pre-test of the survey among 220 residents via the internet and analyzed the data to ensure data quality. Through the initial research, discussion with experts, focus group studies and the pre-test survey we identified and validated the key attributes that are important to Okinawan residents and established a range of values for the amount of money that residents would be willing to contribute for conservation of the Okinawan marine environment through establishment of MPAs.

Based on focus groups and discussions with marine science experts and Okinawan government officials, we identify the following three attributes: 1) leisure fish catch, 2) coral coverage and marine biodiversity, 3) shoreline and coastal conditions, as important components of the Okinawan marine ecosystem. Leisure or recreational fishing is a popular activity in Okinawa [43]https://www.env.go.jp/nature/biodic/coralreefs/reference/contents/0404.pdf and MPAs in Japan are often established with a specific focus on sustaining fishery resources. The coral coverage and marine biodiversity attribute is often the central focus of both centrally managed MPAs that are concerned with preserving the rich coral reef ecosystem and community-based MPAs due to its importance in the local tourism industry. The third attribute, shoreline and coastal conditions, is of major concern for marine conservation because coastal development and reclamation projects affect almost 50% of Okinawan coasts and are responsible for large quantities of red soil run-offs that are adversely affecting the coral reefs in the area.

We also use a fourth attribute, which we call contribution, as the payment instrument that enables us to calculate the WTP. The levels for the first three attributes are stated as expected outcomes 10 years into the future. The contribution is specified as a monthly contribution. We identified the likely range of possible outcomes and also how management actions would impact the levels based on our discussions with government officials and focus group participants. While the levels present plausible outcomes with and without an MPA, the actual levels are general and not based on a scientific study. In Table 1, we provide details and levels of each of the three attributes and contribution that we use for the CE part of the survey. The actual conditions of the status quo (i.e. future scenario without an MPA) leads to a decrease in leisure fish catch and coral coverage and marine biodiversity and an increase in coastal development.

**Table 1:** Details and levels for attributes.

Attribute	Description	Future Possible Levels in 10 Years
Leisure Fish Catch	The average number of fish catch available during a recreational fishing trip after 10 years.	With protected areas: • 30% more fish catch • 15% more fish catch • Current conditions remain Without protected areas (Status Quo): • 15% fewer fish catch
Coral Coverage and Marine Biodiversity	The extent and health of the coral reefs and the number of marine biodiversity found in the Okinawan waters after 10 years.	With protected areas: • 30% more coral coverage and biodiversity • 15% more coral coverage and biodiversity • Current conditions remain Without protected areas (Status Quo): • 15% less coral coverage and biodiversity
Shoreline and Coastal Conditions	The extent of coastal development that includes beachfront construction of homes, hotels, restaurants and roads near or on coastal areas and the condition of the beach and shoreline after 10 years.	With protected areas: • 30% less development with more intact coastal shorelines • 15% less development and moderately intact coastal shoreline • Current conditions remain Without protected areas (Status Quo): • 15% more development with degraded shoreline
Contribution	A monthly contribution will be collected from all Okinawan residents to support the management of these protected areas.	With protected areas: • 100 yen (0.85 USD) per month • 200 yen (1.70 USD) per month • 400 yen (3.40 USD) per month • 600 yen (5.10 USD) per month • 800 yen (6.80 USD) per month • 1000 yen (8.50 USD) per month Without protected areas (Status Quo): • Zero

We follow the standard practice in CE design and use an orthogonal fractional factorial experiment design to select a combination of attributes and levels to design the alternate bundles for the various hypothetical scenarios to present to the respondent [44–46]. The design for the CE was generated using the SAS macro [47] and achieves a 100% D-efficiency and resulted in 81 unique choice questions. The choice questions were blocked into sets of six questions per survey. To account for possible learning that might occur as the respondents are answering the choice questions, we added a dummy question at the beginning of the choice questions and dropped that question from the final analysis [48]. Therefore each respondent answered seven choice questions.

To evaluate the effect of differences in Okinawans’ preferences for community based MPAs versus national based MPAs, we used two different versions of the survey instrument. The two versions were different in only one detail that described who (the central government or the local organizations) would be responsible for identification and management of the MPA referred to in the choice experiment scorecard. Thus, half of the sampled residents were provided with a survey that mentioned that the MPA would be established by the central government whereas the other half were provided with an alternate survey instrument that mentioned that the MPA would be established by participation from local organizations. The respondents were only aware of the management type in their survey and hence would not have known that part of the research questions were about management options. Thus we do not expect strategic bias to have influenced their answers.

In the first section of the survey, we asked the respondents demographic questions to understand what factors may or may not affect choices for specific attributes. In the second section, we provide respondents with seven choice scorecards. For each choice scorecard, the respondent is asked to choose between three possible future scenarios associated with the marine environment. Two of these scenarios have MPAs and one scenario, which represents the status quo, does not have an MPA. In [49], the authors highlight the importance of including this status-quo or opt-out option in CE studies. In the third section of the survey, we asked respondents questions to understand the respondent’s level of involvement with the marine environment in Okinawa. The original survey was conducted in Japanese. We provide examples of the Japanese survey questionnaires and English translations of the survey in supporting information S1–S4 Files.

### Ethics statement

We acquired permission from the OIST Human Subjects Research Review Committee for implementing the survey study. To ensure participation in the survey is voluntary, we provided an explicit statement on the cover letter of the survey document indicating that participation is voluntary. Thus, the fact that the individual decided to voluntarily answer the survey questionnaire after reading this statement in the cover letter provides evidence of consent.

### Implementation

The final survey instrument was administered during December 2014 and January 2015 by Nikkei Research Inc., a professional survey research firm with over 40 years of experience conducting surveys and market research. The survey was conducted via internet among residents of Okinawa over the age of 18. Nikkei Research Inc. sent out the survey questionnaire randomly to individuals from a list of email addresses of residents in Okinawa.

We collected a total of 827 responses from residents of Okinawa of which 422 responded to the national based MPA management regime survey instrument and 405 responded to the community based MPA management regime survey instrument. Table 2 shows the socio-economic characteristics of both sets of respondents that participated in the split-sample CE survey. The first column presents the mean values for the respondents who participated in the national based MPA management regime survey instrument and the second column presents the mean values for the respondents who participated in the community based MPA management regime survey instrument with standard deviations indicated within parenthesis. Based on t-test scores for difference of means between two populations, there is no significant difference between the age groups or gender of respondents that participated in the national based MPA management regime survey versus those who participated in the community based MPA management regime survey.

**Table 2:** Socio-economic characteristics of survey respondents.

	National based MPA management regime Survey Mean (Std. Deviations)	Community based MPA management regime Survey Mean (Std. Deviations)
Gender	0.50 (0.50)	0.52 (0.50)
Age	41.25 (9.78)	41.95 (9.36)
Income Level^a^	3.63 (1.43)	3.70 (1.41)
Born in Okinawa	68% (NA)	70% (NA)
Children under age 18	0.83 (1.13)	0.75 (1.03)

a) Respondents were asked to choose from one of the following five categories to describe their annual income levels: 1) less than $10,000; 2) between $10,000 and $20,000; 3) between $20,000 and $50,000; 4) between $50,000 and $70,000 and 5) more than $70,000.

As shown in Table 2, of the survey respondents, 49% are male and 51% are female. This is relatively similar to the actual ratio of male to female in Okinawa of 48.5 to 51.5. The average age of the respondents is 42 years with a minimum age of 19 years and maximum age of 56 years. This is also close to the actual average age of residents in Okinawa of 41 years. The average income of the survey participants is between 20,000 USD and 50,000 USD. The average per capita income in Okinawa is approximately 20,000 USD. Of the survey respondents, 69% were born in Okinawa and the rest 31% were born outside Okinawa but within Japan. Only three respondents were born outside Japan.

### Model and estimation

We use the mixed multinomial logit (MMNL) model (also referred to as a random parameter logit model) to evaluate the survey responses. The MMNL model is the preferred model for policy analyses because it accounts for preference heterogeneity among the respondents (i.e. it doesn’t assume that the respondents are identical). Previous studies have shown that accounting for individual heterogeneity in preferences is important in prioritizing policy recommendations in the presence of scarce conservation resources and conflicting natural resource management objectives [50].

We use the following main effects specification, which we refer to as Model 1, to estimate the coefficients using the MMNL model:

$$V_{qi}=\beta_{1}*X_{fishcatch}+\beta_{2}*X_{coralcoverage}+\beta_{3}*X_{coastalconditions}+\beta_{4}*X_{payment}+\beta_{5}*X_{ASC}+\varepsilon_{i}$$ (1)

where *V_qi_* is the indirect utility for person *q* from alternative *i*. We also use a second specification, which we refer to as Model 2, in which we include interaction terms for the community-based management, where we define the indirect utility function, *V_qi_* as:

$$\begin{array}{l} V_{qi}=\beta_{1}*X_{fishcatch}+\beta_{2}*X_{coralcoverage}+\beta_{3}*X_{coastalconditions}+\beta_{4}*X_{payment}+\beta_{5}*\\ X_{ASC}+\beta_{6}*S_{community}*X_{fishcatch}+\beta_{7}*S_{community}*X_{coralcoverage}+\beta_{8}*S_{community}*\\ X_{coastalconditions}+\beta_{9}*S_{community}*X_{payment}+\beta_{10}*S_{community}*X_{ASC}+\varepsilon_{i} \end{array}$$ (2)

In Eq 2, *V_qi_* is the indirect utility for person *q* from alternative *i*, and the “*S_community_*” is a dummy variable identifying whether the survey respondent participated in a community based MPA management regime survey.

In both specifications we include an alternative specific constant (ASC) term. The ASC term accounts for the fact that the two MPA options are closer substitutes with each other than the no MPA status quo option [51–52]. We interpret the ASC as identifying the overall preferences for having a MPA irrespective of the attribute values (as opposed to not having an MPA).

We use the coefficient estimates obtained from the main effects specification in Eq 2 to estimate the average value for an additional unit of the selected marine management attribute (*i)*,

$$MWTP_{i}=-\frac{\beta_{i}}{\beta_{payment}}$$ (3)

Finally, we evaluate the MRS between natural capital and man-made capital for establishing MPAs in Okinawa under both, national based and community based, MPA management regimes. Natural capital is a stock of environmental resources which provides a range of goods and services for human use. To estimate the MRS, we use the coral coverage and marine biodiversity attribute as a proxy for natural capital because this attribute provides a wide variety of environmental goods and services for use by residents in Okinawa. Man-made capital refers to the stocks of produced capital such as machines, buildings, infrastructure etc. The attribute coastal and shoreline conditions has been described to survey participants as the extent of coastal development that includes beachfront construction of homes, hotels, restaurants and roads near or on coastal areas. Thus, we use this attribute as a proxy for man-made capital. We use the estimates for coral coverage and marine biodiversity and coastal and shoreline development to evaluate the MRS between natural capital and man-made capital as shown in Eq 4:

$$MRS_{coral\;coverage,coastal\;conditions}=\frac{MU_{coral\;coverage}}{MU_{coastal\;conditions}}$$ (4)

## Results

We present the results for the main effects specification for the MMNL model with the ASC in Table 3. The first column of Table 3 presents the main effects specification for Model 1 (i.e. without the management interactions). The third column presents the main effects specification for Model 2 (i.e. with the management interactions). The second and the fourth columns present the standard deviations for each of the coefficient estimates. We find that the standard deviations for all coefficients are significant indicating that individual heterogeneity is significant for all attributes.

**Table 3:** Regression results for main effects specification.

	Model 1		Model 2	
	Coefficient (SE)	Std. Deviations (SE)	Coefficient (SE)	Std. Deviations (SE)
Fish Catch	0.0069**	0.0259***	0.0035	0.0131*
	(0.0027)	(0.0058)	(0.0035)	(0.0074)
Coral Coverage	0.0360***	0.0435***	0.0299***	0.0367***
	(0.0033)	(0.0055)	(0.0041)	(0.0055)
Coastal Development Restrictions	0.0096***	0.0378***	0.005	-0.0348***
	(0.0029)	(0.0046)	(0.0038)	(0.0049)
Payment	-0.0055***	-0.0051***	-0.0052***	0.0047***
	(0.0003)	(0.0003)	(0.0004)	(0.0003)
ASC	8.220***	-8.521***	8.461***	8.954***
	(0.768)	(0.843)	(0.767)	(0.681)
Fish Catch (Community based MPA)			0.00758	0.0385***
			(0.0058)	(0.0092)
Coral Coverage (Community based MPA)			0.0168**	0.0489***
			(0.0068)	(0.0109)
Coastal Development Restrictions (Community based MPA)			0.0105*	0.0352***
			(0.0061)	(0.0084)
Payment (Community based MPA)			-0.0011**	-0.004***
			(0.0006)	(0.0005)
ASC (Community based MPA)			0.225	-1.131**
			(0.743)	(0.514)
Observations	827		827	

Standard errors are shown in parentheses.

* *p* < 0.10

** *p* < 0.05

*** *p* < 0.01

We discuss the results in detail using the WTP tables in the next section. With regard to the direct coefficient estimations, all three attributes have positive and significant coefficients for the regression based on main effects specification in Model 1. Respondents consider all three attributes as important components of MPAs in Okinawa. The coefficient estimates based on the main effects specification in Model 2 indicate that fish catch is not significant for either set of respondents, both national based MPA management regime and community based MPA management regime. However, we find coastal development to be a significant attribute for respondents who participated in the community based MPA management regime survey instrument. The coral coverage and marine biodiversity attribute is significant for both sets of respondents.

Table 4 shows the valuation of the selected marine management attributes (in yen) for a 1% change in each attribute. Respondents have a higher valuation for an increase in the amount of fish available for catch in ten years, an increase in the extent and health of marine biodiversity in the Okinawan waters and on higher restrictions on coastal development if the MPAs are established by local organizations of Okinawa rather than by the central government. Respondents have a WTP of 17.5 yen (~0.15 USD) for a 10% increase in the number of fish catch under a locally driven, community managed MPA. This is more than 2.5 times what respondents are willing to pay for a similar increase in fish catch under a centrally managed MPA. Both respondents have significant and relatively higher WTP (compared to other attributes) to support increase in the extent and health of coral coverage through MPAs, with a WTP of 73.8 yen (~0.63 USD) and 57.8 yen (~0.50 USD) for a 10% increase in the extent of coral coverage and marine biodiversity through locally managed and centrally managed MPAs, respectively. Thus respondents are willing to pay 28% more for protecting coral coverage and marine biodiversity under a community based management regime. Respondents have a 2.6 times higher WTP for 10% more restriction on coastal development under a locally managed MPA than nationally managed MPAs. The WTP for 10% more coastal restrictions is 95.8 yen (~0.82 USD) for nationally managed MPAs versus 24.8 yen (~0.21 USD) for locally managed MPAs.

**Table 4:** Valuation of the selected marine management attributes.

	Model 1	Model 2
ASC	1489.0***	1631.7***
	(147.8)	(161.8)
Fish Catch	1.244**	0.677
	(0.503)	(0.680)
Coral Coverage	6.524***	5.766***
	(0.616)	(0.821)
Coastal Development Restrictions	1.744***	0.958
	(0.530)	(0.734)
ASC (Community based MPA)		1373.8***
		(137.5)
Fish Catch (Community based MPA)		1.754**
		(0.745)
Coral Coverage (Community based MPA)		7.383***
		(0.920)
Coastal Development Restrictions (Community based MPA)		2.447***
		(0.766)
Observations	827	827

Standard errors in parentheses

* *p* < 0.10

** *p* < 0.05

*** *p* < 0.01

**Fig 1:**
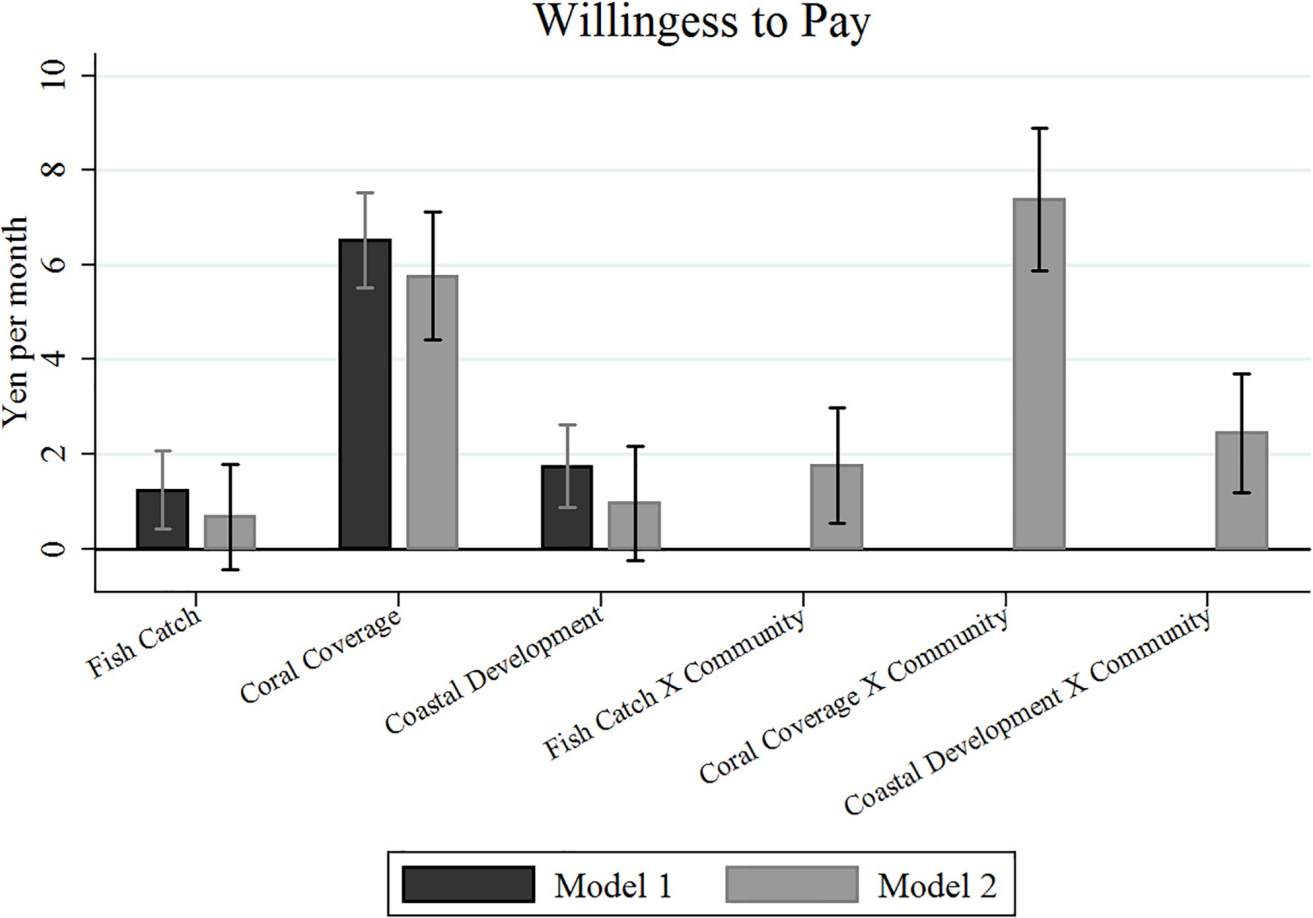
Valuation of the selected marine management attributes.

Results shown in Table 4 and Fig 1 highlight our key finding that the valuation of the selected attributes are resilient to the two econometric model specifications, and this supports the scientific robustness of the proposed values for policy guidance.

We present the results for the MRS between natural capital and man-made capital in Table 5. The extent and health of coral coverage and marine biodiversity represents natural capital and the coastal and shoreline development represents man-made capital. According to the survey, respondents are willing to give up six unit of man-made capital to gain one unit of natural capital under a national based MPA management regime. On the other hand, respondents are willing to trade-off three units of man-made capital to gain one unit of natural capital under a community based MPA management regime. According to these results, natural capital is seen as the most valued form of capital relative to man-made capital irrespective of the management regime under consideration. However, this relative value of natural capital is higher under a national based MPA management regime.

**Table 5:** Marginal rate of substitution between natural capital and man-made capital for MPAs with top down and bottom-up management regimes.

	MRS _coral coverage, coastal conditions_
Top Down Management Regime	6.021
	(4.660)
Bottom-up Management Regime	3.017
	(0.976)

Standard errors shown in parentheses

## Discussion and conclusion

Respondents favor MPAs that are centered on the extension of the coral coverage along the coast of Okinawa, which in this experiment is interpreted as the conservation of marine environment, regardless of the management types. Thus, emphasizing these benefits when proposing and planning protected areas may garner more support for marine protection, irrespective of the management plan under consideration. However, even for the marine conservation attribute, respondents have a higher level of support when the MPA design and management involve local, coastal communities. This informs that the level of respect and trust that respondents place on the local, coastal communities when managing marine conservation is particularly strong when the proposed objective of the MPA is anchored in the marine conservation and coastal development restrictions attribute.

Based on our valuation results, we did not find an increase in leisure fish catch to be a significant attribute associated with respondent’s choice of MPAs in Okinawa. This result was initially surprising but upon further analyzing the sample we find that most of the sample does not engage in recreational fishing (91% disagreed with the statement “they regularly engage in recreational fishing”). Given that many of the respondents in the sample do not engage in recreational fishing it is not surprising that the fish catch is not significant. At the same time, there are growing concerns over the steadily declining commercial fish catch across Japan [53]. Thus, future studies in the region can benefit from using targeted surveys that collect information from local commercial and recreational fishermen who are key stakeholders in the marine environment of Okinawa.

We find that the overall value of the proposed MPA, and its composed attributes, is higher in a community based MPA management regime. This further underpins the importance of the management regime of the proposed MPA. Our results indicate that there is a significant and large difference in the willingness of Okinawan residents to support MPA efforts in the area depending on whether local or central organization(s) are involved in the designing and administration of the instituted MPAs. Thus, by whom and how the MPA is governed is an important feature and will likely influence the initial support for and the ultimate success or failure of MPAs established in Okinawa. This finding complements the findings in [13].

Our results show that respondents are willing to support more marine management attributes with a higher WTP for each of those attributes when the MPA management regime is community based. Thus, our results strengthen the importance and necessity of a more collaborative approach towards MPA designation and management that involve participation from local communities and stakeholders. Studies have shown that conservation planners are in favor of including local stakeholders and communities in the management process [54] and that conservation donors have a higher willingness to pay for projects with community involvement [55]. Other studies find that co-management or a mixed management approach to MPAs can provide a much needed compromise between strictly community-based or bottom-up MPAs and national based or top-down MPAs [56–59]. Such co-managed MPAs can provide a win-win solution as they can combine the local knowledge and expertise of stakeholders and communities that will be most directly affected by the MPA with the resources and governing authority of the central government.

Finally, estimation results inform that residents of Okinawa see natural capital as the most valued form of capital. However, the relative difference between natural capital and man-made capital depends on the proposed management regime. According to the results, the relative value of natural capital is higher under a national based management regime compared to community based management regime. Thus, residents under a community-based MPA management regime need to be compensated relatively less in man-made capital in the presence of a policy scenario that proposes an increase in natural capital. These results suggest that increasing the present area under MPA in Okinawa that mainly focuses on marine conservation–in particular increasing and protecting the coral area–presents lower opportunity costs of the public money invested if the proposed policy scenario, and underlying management scheme, is done by local coastal communities.

Our results provide significant information to the policy maker. From an efficiency perspective, these results suggest that increasing the present area under MPA in Okinawa that mainly focuses on marine conservation–in particular increasing and protecting the coral area–presents lower opportunity costs of the public money invested if the proposed management regime is done by local coastal communities and will have the full support of the residents of Okinawa. However, given some of the advantages of nationally-managed MPA programs, Okinawan residents will be much more supportive of such top-down management regimes when the proposed MPA focuses on maintaining and enhancing the coral coverage and conservation of marine biodiversity along the coast of Okinawa.

## Supporting information

S1 File Survey Questionnaire (centrally-managed MPA management regime) in Japanese. This pdf file shows the actual online survey document that was used by Nikkei Research Inc. to collect data from residents in Okinawa over the age of 18. The survey in this pdf represents the centrally-managed MPA regime.
(PDF)

S2 File Survey Questionnaire (community-based MPA management regime) in Japanese. This pdf file shows the actual online survey document that was used by Nikkei Research Inc. to collect data from residents in Okinawa over the age of 18. The survey in this pdf represents the community-managed MPA regime.
(PDF)

S3 File English Translation of Survey Questionnaire (centrally-managed MPA management regime). This pdf file provides an English translation of the centrally-managed MPA management regime survey document. In the translated version, we only include one choice scorecard. However, in the actual survey each respondent was provided with seven choice scorecards.
(PDF)

S4 File English Translation of Survey Questionnaire (community-based MPA management regime). This pdf file provides an English translation of the community-based MPA management regime survey document. In the translated version, we only include one choice scorecard. However, in the actual survey each respondent was provided with seven choice scorecards.
(PDF)
