# Supplementary material for: Impact of a local, coastal community based management regime when defining marine protected areas: Empirical results from a study in Okinawa, Japan
Source: PLoS One. 2019 Mar 8;14(3):e0213354. doi: 10.1371/journal.pone.0213354 (PMC6407762; doi:10.1371/journal.pone.0213354)
Supplement: S2 File — This pdf file shows the actual online survey document that was used by Nikkei Research Inc. to collect data from residents in Okinawa over the age of 18. The survey in this pdf represents the community-managed MPA regime. (PDF) [file pone.0213354.s002.pdf]

沖縄の海洋環境に関する意識調査

start　end

小 中 大
文字サイズ

再ログイン時には下記ＩＤ番号、パスワードが必要です。
＜ＩＤ番号、パスワードをメモしておくことをお薦めします＞

ＩＤ番号:	
パスワード:	

【回答締め切り】

2014年12月12日(金) 13:00

【実施会社】

株式会社日経リサーチ
〒101-0047　東京都千代田区内神田2-2-1 鎌倉河岸ビル
担当：稲村

【個人情報の取扱いについて】

当社は、一般財団法人日本情報経済社会推進協会より、個人情報の適切な取り扱いを行う事業者に付与されるプライバシーマークの付与認定を受けています。
当社の個人情報保護方針については、日経リサーチ社WEBサイトの個人情報保護方針をご参照下さい。
弊社の個人情報保護管理者は取締役　鈴木督久です。

お答えいただいたアンケートの結果はコンピューターで統計的に処理し、「○○と回答した人が何%」「男性の支持は高いが、女性の支持は低い」というような統計値として使用します。
従って、事後にＤＭ（ダイレクトメール）を送りつけたり、営業に訪問するなどご回答者様にご迷惑をおかけすることは決してありません。

たいせつにしますプライバシー
12390037(08)

【お問い合わせについて】

本調査に関するお問い合わせは[i_monitor@nikkei-r.co.jp] までお願いいたします。

アンケートへ ▶

- 回答内容により質問番号が飛ぶことがありますが、気にせずにご回答ください。
- 回線の混雑などが原因で反応が遅くなることがあります。ご了承ください。
- ブラウザの戻るボタンで戻った場合、回答が正しく受け付けられない場合があります。その場合、再度アンケートを最初からやり直して下さい。

(調査NO. 14-406-0478)

NIKKEI-R

【当ＷＥＢシステム利用上の注意】

再ログイン時には下記ＩＤ番号、パスワードが必要です。
＜ＩＤ番号、パスワードをメモしておくことをお薦めします＞

ＩＤ番号:	
パスワード:	

- 回答中に前ページに戻る際には、ページの一番下の「戻る」ボタンをクリックしてください。ブラウザの「戻る」ボタンは使用しないようご注意ください。
- 回答を途中で中断し、後で再開することも可能です。
  中断する場合は、ページの一番下の「一時保存をして中断」ボタンをクリックして下さい。
- アンケートを再開される場合は、依頼メールに記載されているURLを再度クリックしていただくか、マイページの本アンケートへのリンクをクリックしてください。中断した箇所よりアンケートを再開します。
- 回答期間中は、土日も含め24時間回答可能です。
- 推奨ブラウザはWindowsでは「InternetExplorer6～10」です。これ以外のブラウザでは回答時にエラーが出る可能性があります。
- 一度、回答を完了すると再回答はできませんのでご注意下さい。
- 回線の混雑などが原因で反応が遅くなることがあります。ご了承ください。

次へ ▶

調査の背景と目的：

沖縄の海は、レクリエーション、海岸の保護、魚や貝などの海洋生物の生息場所の保護といった、市場で売買されることのない多くの恩恵を生み出しています。沖縄の環境や経済や社会の豊かさは、これらの多様な恩恵によりもたらされています。しかしながら、沖縄の海洋生態系は、気候変動や人間の活動による数々の脅威にさらされています。これらの脅威を防ぐ方法の一つに、保護区を設置するという解決策があります。保護区内の海洋環境は周囲の地域に比べ、保全が強化されます。この保全により、海洋環境は過去のダメージから回復し、今後のダメージも緩和できます。本調査の目的は、沖縄の海洋環境から生み出される（市場取引の対象とはならないような）重要な特性について、人々がどのように考えているかを明らかにすることにあります。

調査の内容と方法：

本調査は3部で構成されています。第1部では、あなたご自身に関していくつかの短い質問をいたします。第2部では、いろいろな海洋保護政策とその効果を仮定して示しますので、その中から最も好ましいものを1つお選びいただきます。それぞれの選択肢は海から得られる3種類の恩恵の組み合わせで構成されていますが、その中からあなたが最も好ましいと思うものを1つ選んでいただきます。第3部では、沖縄の海洋環境に対するあなたの関わり方を確認するための質問をいたします。

回答内容は本調査の分析以外には一切利用いたしませんのでご安心ください。

一時保存をして中断

次へ

- 回答内容により質問番号が飛ぶことがありますが、気にせずにご回答ください。
- 回線の混雑などが原因で反応が遅くなることがあります。ご了承ください。
- ブラウザの戻るボタンで戻った場合、回答が正しく受け付けられない場合があります。その場合、再度アンケートを最初からやり直して下さい。

沖縄の海洋環境に関する意識調査

start end 小 中 大 文字サイズ

第1部

ここではあなたご自身のことについて伺いますが、個人情報は他に漏れることのないよう厳重に管理し、本調査の分析・考察以外には一切用いません。

一時保存をして中断

次へ

- 回答内容により質問番号が飛ぶことがありますが、気にせずにご回答ください。
- 回線の混雑などが原因で反応が遅くなることがあります。ご了承ください。
- ブラウザの戻るボタンで戻った場合、回答が正しく受け付けられない場合があります。その場合、再度アンケートを最初からやり直して下さい。

(調査NO. 14-406-0478)

NIKKEI-R

沖縄の海洋環境に関する意識調査

start end 文字サイズ 小 中 大

F1

ご自身またはあなたの世帯に、漁業に関係する職業に就いている方はいますか。

（ひとつだけ）

- ○ はい
- ○ いいえ

F2

ご友人に漁業に関係する職業に就いている方はいますか。

（ひとつだけ）

- ○ はい
- ○ いいえ

F3

ご自身またはあなたのご家族で海のレジャーに関係する職業に就いている方はいますか。

（ひとつだけ）

- ○ はい
- ○ いいえ

F4

あなたのご友人で、海のレジャーに関係する職業に就いている方はいますか。

（ひとつだけ）

- ○ はい
- ○ いいえ

▶ このページに入力したものを全てクリアする

▶ 一時保存をして中断

次へ ▶

- 回答内容により質問番号が飛ぶことがありますが、気にせずにご回答ください。
- 回線の混雑などが原因で反応が遅くなることがあります。ご了承ください。
- ブラウザの戻るボタンで戻った場合、回答が正しく受け付けられない場合があります。その場合、再度アンケートを最初からやり直して下さい。

（調査NO. 14-406-0478）

NIKKEI-R

F5

ご自身に当てはまるものはどれですか。

（ひとつだけ）

- フルタイム勤務
- パートタイム勤務
- 自営業
- 全日制の学生
- 定時制の学生
- 専業主婦・主夫
- 退職者
- 無職
- その他 （具体的に： ）

このページに入力したものを全てクリアする

一時保存をして中断

次へ

- 回答内容により質問番号が飛ぶことがありますが、気にせずにご回答ください。
- 回線の混雑などが原因で反応が遅くなることがあります。ご了承ください。
- ブラウザの戻るボタンで戻った場合、回答が正しく受け付けられない場合があります。その場合、再度アンケートを最初からやり直して下さい。

沖縄の海洋環境に関する意識調査

start end

小 中 大 文字サイズ

F6

お住まいの市町村の郵便番号をご入力ください。

－	－	－	－	－	－	－
0	0	0	0	0	0	0
1	1	1	1	1	1	1
2	2	2	2	2	2	2
3	3	3	3	3	3	3
4	4	4	4	4	4	4
5	5	5	5	5	5	5
6	6	6	6	6	6	6
7	7	7	7	7	7	7
8	8	8	8	8	8	8
9	9	9	9	9	9	9

F7

あなたの世帯に18歳未満の方は何人いますか。

（ひとつだけ）

▽以下から選択
0人
1人
2人
3人
4人
5人
6人
7人
8人
9人
10人以上

F8

あなたの性別をお答えください。

（ひとつだけ）

- ○ 男性
- ○ 女性

F9

あなたの年齢をお答えください。

（ひとつだけ）

▽以下から選択
18歳
19歳
20歳
21歳
22歳
23歳
24歳
25歳
26歳
27歳
28歳
29歳
30歳
31歳
32歳
33歳
34歳
35歳
36歳
37歳
38歳
39歳
40歳
41歳
42歳
43歳
44歳
45歳
46歳
47歳
48歳
49歳
50歳
51歳
52歳
53歳
54歳
55歳
56歳以上

▶ このページに入力したものを全てクリアする

▶ 一時保存をして中断

次へ ▶

- 回答内容により質問番号が飛ぶことがありますが、気にせずにご回答ください。
- 回線の混雑などが原因で反応が遅くなることがあります。ご了承ください。
- ブラウザの戻るボタンで戻った場合、回答が正しく受け付けられない場合があります。その場合、再度アンケートを最初からやり直して下さい。

（調査NO. 14-406-0478）

NIKKEI-R

F10

あなたの世帯の年間収入に最も近いものをお選びください。 （ひとつだけ）

- 100万円未満
- 100～200万円未満
- 200～500万円未満
- 500～700万円未満
- 700万円以上
- わからない/答えたくない

F11

出身地はどちらですか。 （ひとつだけ）

- 沖縄県
- 沖縄県外
- 海外

このページに入力したものを全てクリアする

一時保存をして中断

次へ

- 回答内容により質問番号が飛ぶことがありますが、気にせずにご回答ください。
- 回線の混雑などが原因で反応が遅くなることがあります。ご了承ください。
- ブラウザの戻るボタンで戻った場合、回答が正しく受け付けられない場合があります。その場合、再度アンケートを最初からやり直して下さい。

第2部

次に沖縄の海洋生態系を守るために、【沖縄の地域団体で】複数の海洋保護区を設置されると仮定します。海洋保護区を設定した場合、そこで取られる保護策の水準によって下表に示したような様々な効果が予想されます。下表をよくご覧になった後、次の質問に進んでください。

保護策の影響を受ける特性	保護策の効果の水準を示す指標	現状（保全策をとる前のイメージ）	保護区において10年後に予測される状態の選択肢
漁獲量	10年後のレジャーとしての釣りによって釣られる平均的な魚の量	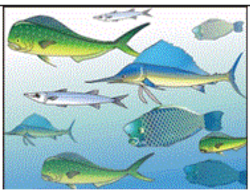	保護区がある場合：<br>1) 30%増加<br>2) 15%増加<br>3) 現在の状態のまま<br>保護区が無い場合：<br>1) 15%減少
サンゴ被度と海洋の生物多様性	10年後の沖縄の海洋におけるサンゴ礁の量およびその健康状態と生物の多様性<br>※サンゴ礁は多くの生物の生息場所となります。サンゴ被度（海底面をサンゴが被っている面積の割合）が高い場合、サンゴ礁が健康であり、多様な海洋生物が生息していることを示しています。	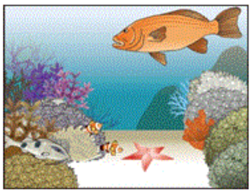	保護区がある場合：<br>1) サンゴ被度および生物多様性は30%増加<br>2) サンゴ被度および生物多様性は15%増加<br>3) 現在の状態のまま<br>保護区が無い場合：<br>1) サンゴ被度および生物多様性は15%減少
海岸線および沿岸部の状態	沿岸およびその近隣における住宅・宿泊施設・飲食店・道路の建設など、10年後の沖縄の沿岸開発の程度および浜辺や海岸線の状況	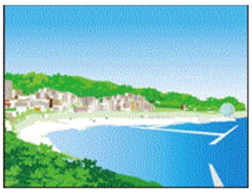	保護区がある場合：<br>1) 開発を30%とりやめ、沿岸部の海岸線を保全する<br>2) 開発を15%とりやめ、沿岸部の海岸線を多少保全する<br>3) 現在の状態のまま<br>保護区が無い場合：<br>1) 海岸線が損傷しても15%開発を増やす
寄付金	これらの保護区管理を支援するために、沖縄県民全員から毎月寄付金を募る。		保護区がある場合：<br>1) 月100円<br>2) 月200円<br>3) 月400円<br>4) 月600円<br>5) 月800円<br>6) 月1000円<br>保護区が無い場合：<br>1) なし

一時保存をして中断

次へ

沖縄の海洋環境に関する意識調査

start end

小 中 大
文字サイズ

これから７つの異なる質問が表示されます。 各質問では、海洋環境に関する解説をよくご覧の上、お答えください。

一時保存をして中断

次へ

- 回答内容により質問番号が飛ぶことがありますが、気にせずにご回答ください。
- 回線の混雑などが原因で反応が遅くなることがあります。ご了承ください。
- ブラウザの戻るボタンで戻った場合、回答が正しく受け付けられない場合があります。その場合、再度アンケートを最初からやり直して下さい。

(調査NO. 14-406-0478)

NIKKEI-R

Q17

これらの監視と管理は、あなたの寄付金によって賄われると仮定します。以下の3つの選択肢のうち、1つを選ぶと考えてください。将来のシナリオAとBは、海洋保護区が設置された場合に考えられる10年後の状態です。将来のシナリオCは、沖縄に海洋保護区が設置されなかった場合に考えられえる今から10年後の状態です。あなたにとって最も好ましいのはシナリオA～Cのうちどれですか。

（ひとつだけ）

	将来のシナリオA<br>10年後に予測される状態	将来のシナリオB<br>10年後に予測される状態	将来のシナリオC<br>10年後に予想される状態（保護方針がない場合）
10年後のレジャーとしての釣りによって釣られる平均的な魚の量	現在の状態のまま<br>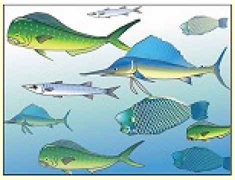	15%増加<br>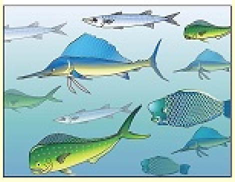	15%減少<br>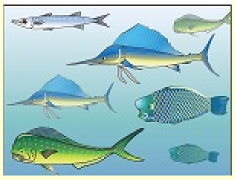
サンゴ被度と海洋の生物多様性	サンゴ被度および生物多様性は30%増加<br>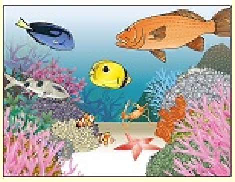	サンゴ被度および生物多様性は15%増加<br>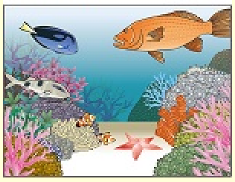	サンゴ被度および生物多様性は15%減少<br>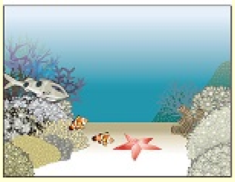
海岸線および沿岸部の状態	現在の状態のまま<br>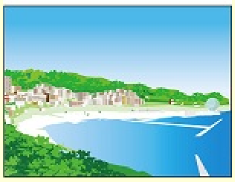	開発を30%とりやめ、沿岸部の海岸線を保全する<br>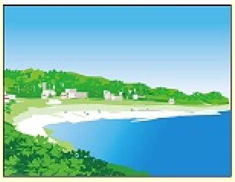	海岸線が損傷しても15%開発を増やす<br>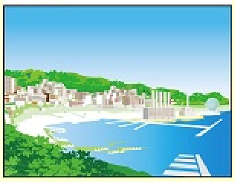
あなたが支払う寄付金額	月600円	月200円	なし
	↓	↓	↓
私の選択は	○	○	○

▶ このページに入力したものを全てクリアする

▶ 一時保存をして中断

次へ ▶

- 回答内容により質問番号が飛ぶことがありますが、気にせずにご回答ください。
- 回線の混雑などが原因で反応が遅くなることがあります。ご了承ください。
- ブラウザの戻るボタンで戻った場合、回答が正しく受け付けられない場合があります。その場合、再度アンケートを最初からやり直して下さい。

Q18

これらの監視と管理は、あなたの寄付金によって賄われると仮定します。以下の3つの選択肢のうち、1つを選ぶと考えてください。将来のシナリオAとBは、海洋保護区が設置された場合に考えられる10年後の状態です。将来のシナリオCは、沖縄に海洋保護区が設置されなかった場合に考えられえる今から10年後の状態です。あなたにとって最も好ましいのはシナリオA～Cのうちどれですか。

（ひとつだけ）

	将来のシナリオA<br>10年後に予測される状態	将来のシナリオB<br>10年後に予測される状態	将来のシナリオC<br>10年後に予想される状態<br>（保護方針がない場合）
10年後のレジャーとしての釣りによって釣られる平均的な魚の量	現在の状態のまま 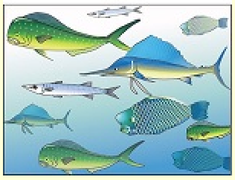	30％増加 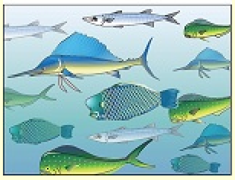	15％減少 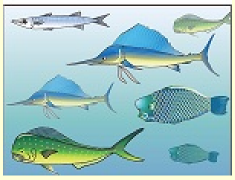
サンゴ被度と海洋の生物多様性	サンゴ被度および生物多様性は15％増加 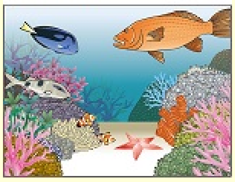	現在の状態のまま 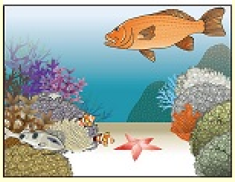	サンゴ被度および生物多様性は15％減少 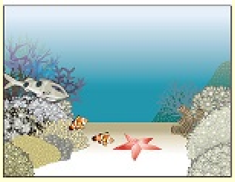
海岸線および沿岸部の状態	開発を30％とりやめ、沿岸部の海岸線を保全する 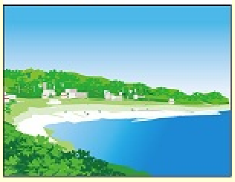	開発を15％とりやめ、沿岸部の海岸線を多少保全する 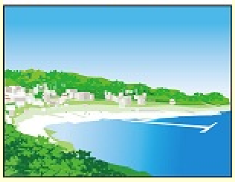	海岸線が損傷しても15％開発を増やす 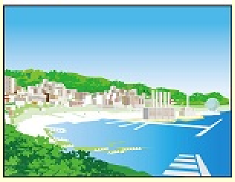
あなたが支払う寄付金額	月800円	月1000円	なし
	↓	↓	↓
私の選択は	○	○	○

▶ このページに入力したものを全てクリアする

▶ 一時保存をして中断

次へ ▶

- 回答内容により質問番号が飛ぶことがありますが、気にせずにご回答ください。
- 回線の混雑などが原因で反応が遅くなることがあります。ご了承ください。
- ブラウザの戻るボタンで戻った場合、回答が正しく受け付けられない場合があります。その場合、再度アンケートを最初からやり直して下さい。

Q19

これらの監視と管理は、あなたの寄付金によって賄われると仮定します。以下の3つの選択肢のうち、1つを選ぶと考えてください。将来のシナリオAとBは、海洋保護区が設置された場合に考えられる10年後の状態です。将来のシナリオCは、沖縄に海洋保護区が設置されなかった場合に考えられえる今から10年後の状態です。あなたにとって最も好ましいのはシナリオA～Cのうちどれですか。

（ひとつだけ）

	将来のシナリオA<br>10年後に予測される状態	将来のシナリオB<br>10年後に予測される状態	将来のシナリオC<br>10年後に予想される状態<br>（保護方針がない場合）
10年後のレジャーとしての釣りによって釣られる平均的な魚の量	15%増加<br>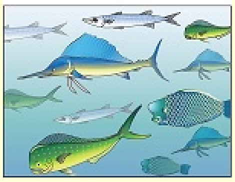	現在の状態のまま<br>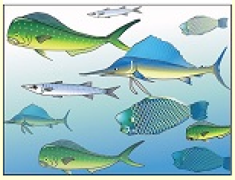	15%減少<br>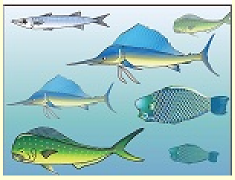
サンゴ被度と海洋の生物多様性	サンゴ被度および生物多様性は30%増加<br>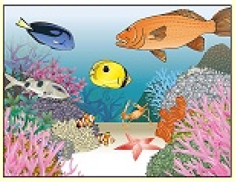	現在の状態のまま<br>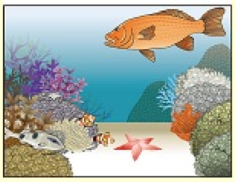	サンゴ被度および生物多様性は15%減少<br>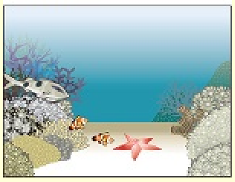
海岸線および沿岸部の状態	現在の状態のまま<br>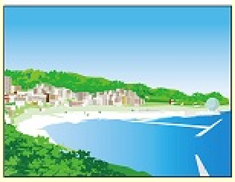	開発を15%とりやめ、沿岸部の海岸線を多少保全する<br>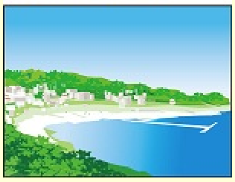	海岸線が損傷しても15%開発を増やす<br>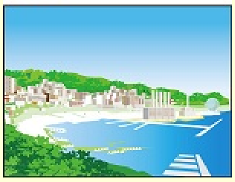
あなたが支払う寄付金額	月200円	月100円	なし
	↓	↓	↓
私の選択は	○	○	○

▶ このページに入力したものを全てクリアする

▶ 一時保存をして中断

次へ ▶

- 回答内容により質問番号が飛ぶことがありますが、気にせずにご回答ください。
- 回線の混雑などが原因で反応が遅くなることがあります。ご了承ください。
- ブラウザの戻るボタンで戻った場合、回答が正しく受け付けられない場合があります。その場合、再度アンケートを最初からやり直して下さい。

Q20

これらの監視と管理は、あなたの寄付金によって賄われると仮定します。以下の3つの選択肢のうち、1つを選ぶと考えてください。将来のシナリオAとBは、海洋保護区が設置された場合に考えられる10年後の状態です。将来のシナリオCは、沖縄に海洋保護区が設置されなかった場合に考えられえる今から10年後の状態です。あなたにとって最も好ましいのはシナリオA～Cのうちどれですか。

（ひとつだけ）

	将来のシナリオA<br>10年後に予測される状態	将来のシナリオB<br>10年後に予測される状態	将来のシナリオC<br>10年後に予想される状態<br>（保護方針がない場合）
10年後のレジャーとしての釣りによって釣られる平均的な魚の量	15%増加<br>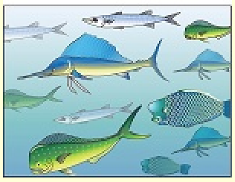	30%増加<br>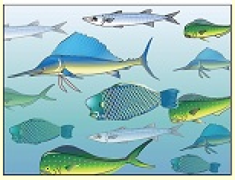	15%減少<br>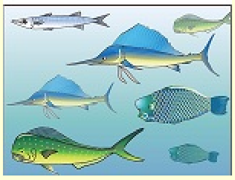
サンゴ被度と海洋の生物多様性	現在の状態のまま<br>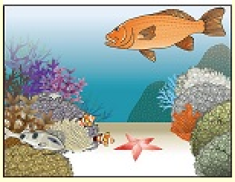	サンゴ被度および生物多様性は30%増加<br>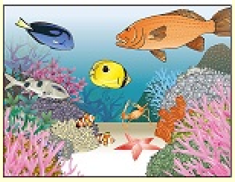	サンゴ被度および生物多様性は15%減少<br>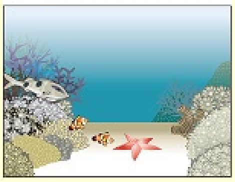
海岸線および沿岸部の状態	開発を30%とりやめ、沿岸部の海岸線を保全する<br>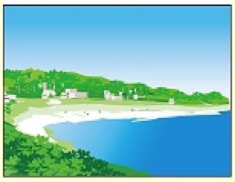	開発を15%とりやめ、沿岸部の海岸線を多少保全する<br>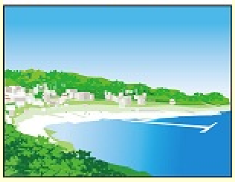	海岸線が損傷しても15%開発を増やす<br>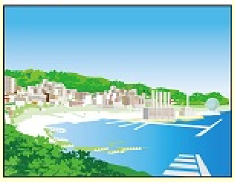
あなたが支払う寄付金額	月600円	月200円	なし
	↓	↓	↓
私の選択は	○	○	○

このページに入力したものを全てクリアする

一時保存をして中断

次へ

- 回答内容により質問番号が飛ぶことがありますが、気にせずにご回答ください。
- 回線の混雑などが原因で反応が遅くなることがあります。ご了承ください。
- ブラウザの戻るボタンで戻った場合、回答が正しく受け付けられない場合があります。その場合、再度アンケートを最初からやり直して下さい。

（調査NO. 14-406-0478）

Q21

これらの監視と管理は、あなたの寄付金によって賄われると仮定します。以下の3つの選択肢のうち、1つを選ぶと考えてください。将来のシナリオAとBは、海洋保護区が設置された場合に考えられる10年後の状態です。将来のシナリオCは、沖縄に海洋保護区が設置されなかった場合に考えられえる今から10年後の状態です。あなたにとって最も好ましいのはシナリオA～Cのうちどれですか。

（ひとつだけ）

	将来のシナリオA<br>10年後に予測される状態	将来のシナリオB<br>10年後に予測される状態	将来のシナリオC<br>10年後に予想される状態<br>（保護方針がない場合）
10年後のレジャーとしての釣りによって釣られる平均的な魚の量	30%増加<br>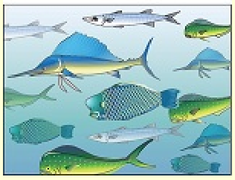	15%増加<br>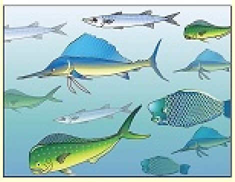	15%減少<br>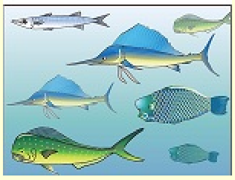
サンゴ被度と海洋の生物多様性	サンゴ被度および生物多様性は30%増加<br>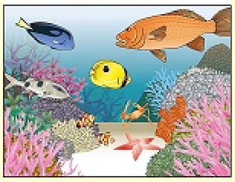	現在の状態のまま<br>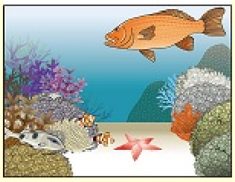	サンゴ被度および生物多様性は15%減少<br>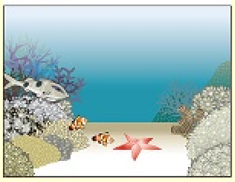
海岸線および沿岸部の状態	開発を30%とりやめ、沿岸部の海岸線を保全する<br>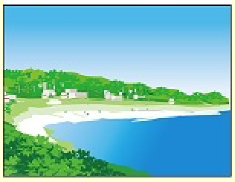	現在の状態のまま<br>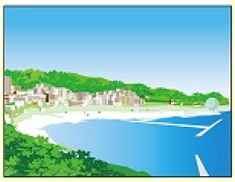	海岸線が損傷しても15%開発を増やす<br>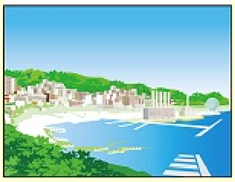
あなたが支払う寄付金額	月800円	月200円	なし
	↓	↓	↓
私の選択は	○	○	○

▶ このページに入力したものを全てクリアする

▶ 一時保存をして中断

次へ ▶

- 回答内容により質問番号が飛ぶことがありますが、気にせずにご回答ください。
- 回線の混雑などが原因で反応が遅くなることがあります。ご了承ください。
- ブラウザの戻るボタンで戻った場合、回答が正しく受け付けられない場合があります。その場合、再度アンケートを最初からやり直して下さい。

NIKKEI-R

（調査NO. 14-406-0478）

沖縄の海洋環境に関する意識調査

start end

小 中 大 文字サイズ

Q22

これらの監視と管理は、あなたの寄付金によって賄われると仮定します。以下の3つの選択肢のうち、1つを選ぶと考えてください。将来のシナリオAとBは、海洋保護区が設置された場合に考えられる10年後の状態です。将来のシナリオCは、沖縄に海洋保護区が設置されなかった場合に考えられえる今から10年後の状態です。あなたにとって最も好ましいのはシナリオA～Cのうちどれですか。

（ひとつだけ）

	**将来のシナリオA** 10年後に予測される状態	**将来のシナリオB** 10年後に予測される状態	**将来のシナリオC** 10年後に予想される状態（保護方針がない場合）
10年後のレジャーとしての釣りによって釣られる平均的な魚の量	**30%増加** 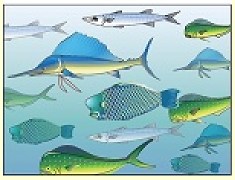	**現在の状態のまま** 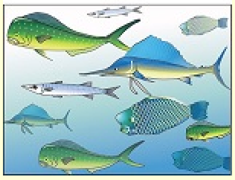	**15%減少** 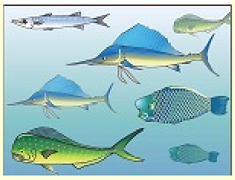
サンゴ被度と海洋の生物多様性	**サンゴ被度および生物多様性は15%増加** 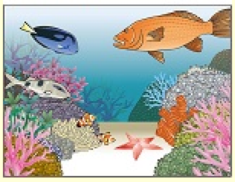	**現在の状態のまま** 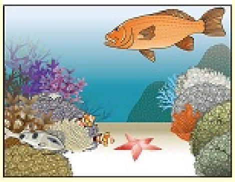	**サンゴ被度および生物多様性は15%減少** 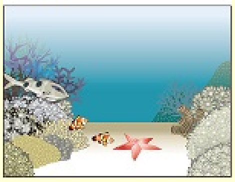
海岸線および沿岸部の状態	**開発を30%とりやめ、沿岸部の海岸線を保全する** 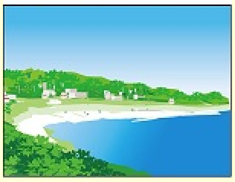	**開発を15%とりやめ、沿岸部の海岸線を多少保全する** 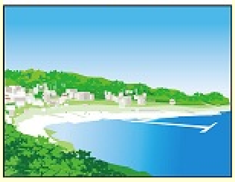	**海岸線が損傷しても15%開発を増やす** 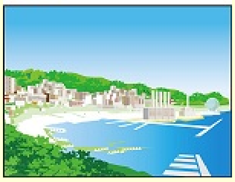
あなたが支払う寄付金額	月800円	月600円	なし
	↓	↓	↓
私の選択は	○	○	○

▶ このページに入力したものを全てクリアする

▶ 一時保存をして中断

次へ ▶

- 回答内容により質問番号が飛ぶことがありますが、気にせずにご回答ください。
- 回線の混雑などが原因で反応が遅くなることがあります。ご了承ください。
- ブラウザの戻るボタンで戻った場合、回答が正しく受け付けられない場合があります。その場合、再度アンケートを最初からやり直して下さい。

（調査NO. 14-406-0478）

NIKKEI-R

Q23

これらの監視と管理は、あなたの寄付金によって賄われると仮定します。以下の3つの選択肢のうち、1つを選ぶと考えてください。将来のシナリオAとBは、海洋保護区が設置された場合に考えられる10年後の状態です。将来のシナリオCは、沖縄に海洋保護区が設置されなかった場合に考えられえる今から10年後の状態です。あなたにとって最も好ましいのはシナリオA～Cのうちどれですか。

（ひとつだけ）

	将来のシナリオA<br>10年後に予測される状態	将来のシナリオB<br>10年後に予測される状態	将来のシナリオC<br>10年後に予想される状態<br>（保護方針がない場合）
10年後のレジャーとしての釣りによって釣られる平均的な魚の量	現在の状態のまま<br>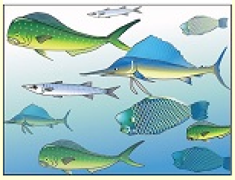	15%増加<br>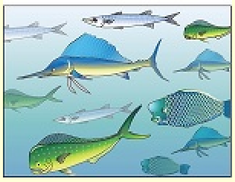	15%減少<br>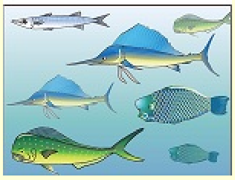
サンゴ被度と海洋の生物多様性	サンゴ被度および生物多様性は30%増加<br>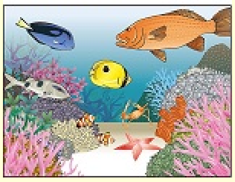	サンゴ被度および生物多様性は15%増加<br>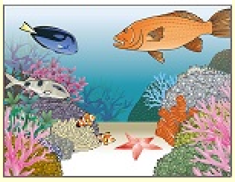	サンゴ被度および生物多様性は15%減少<br>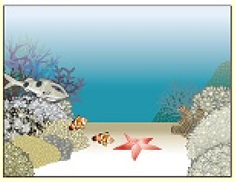
海岸線および沿岸部の状態	現在の状態のまま<br>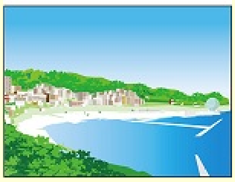	開発を30%とりやめ、沿岸部の海岸線を保全する<br>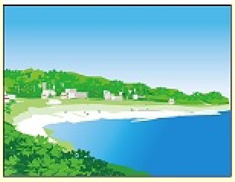	海岸線が損傷しても15%開発を増やす<br>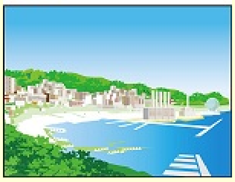
あなたが支払う寄付金額	月600円	月200円	なし
	↓	↓	↓
私の選択は	○	○	○

▶ このページに入力したものを全てクリアする

▶ 一時保存をして中断

次へ ▶

- 回答内容により質問番号が飛ぶことがありますが、気にせずにご回答ください。
- 回線の混雑などが原因で反応が遅くなることがあります。ご了承ください。
- ブラウザの戻るボタンで戻った場合、回答が正しく受け付けられない場合があります。その場合、再度アンケートを最初からやり直して下さい。

（調査NO. 14-406-0478）

NIKKEI-R

第3部

Q1

私の家族は、少なくとも週1回魚介類を食べる。 (ひとつだけ)

- ○ 非常にあてはまる
- ○ あてはまる
- ○ どちらともいえない
- ○ あてはまらない
- ○ 全くあてはまらない

Q2

私の家族は、少なくとも月1回魚介類を食べる。 (ひとつだけ)

- ○ 非常にあてはまる
- ○ あてはまる
- ○ どちらともいえない
- ○ あてはまらない
- ○ 全くあてはまらない

- 回答内容により質問番号が飛ぶことがありますが、気にせずにご回答ください。
- 回線の混雑などが原因で反応が遅くなることがあります。ご了承ください。
- ブラウザの戻るボタンで戻った場合、回答が正しく受け付けられない場合があります。その場合、再度アンケートを最初からやり直して下さい。

(調査NO. 14-406-0478)

沖縄の海洋環境に関する意識調査

start end

小 中 大 文字サイズ

Q3

私は週に1度はレジャーとして釣りをしている。

（ひとつだけ）

- ○ 非常にあてはまる
- ○ あてはまる
- ○ どちらともいえない
- ○ あてはまらない
- ○ 全くあてはまらない

▶ このページに入力したものを全てクリアする

▶ 一時保存をして中断

次へ ▶

- 回答内容により質問番号が飛ぶことがありますが、気にせずにご回答ください。
- 回線の混雑などが原因で反応が遅くなることがあります。ご了承ください。
- ブラウザの戻るボタンで戻った場合、回答が正しく受け付けられない場合があります。その場合、再度アンケートを最初からやり直して下さい。

（調査NO. 14-406-0478）

NIKKEI-R

Q4

家で食べる魚介類は、私か家族が採ってくる。 （ひとつだけ）

- 非常にあてはまる
- あてはまる
- どちらともいえない
- あてはまらない
- 全くあてはまらない

Q5

家で食べる魚介類は、地元の市場で購入するものである。 （ひとつだけ）

- 非常にあてはまる
- あてはまる
- どちらともいえない
- あてはまらない
- 全くあてはまらない

Q6

通常、私と家族は沖縄の海で採れた魚介類を食べている。 （ひとつだけ）

- 非常にあてはまる
- あてはまる
- どちらともいえない
- あてはまらない
- 全くあてはまらない

Q7

通常、私と家族は沖縄以外で採れた魚介類を食べている。 （ひとつだけ）

- 非常にあてはまる
- あてはまる
- どちらともいえない
- あてはまらない
- 全くあてはまらない

- 回答内容により質問番号が飛ぶことがありますが、気にせずにご回答ください。
- 回線の混雑などが原因で反応が遅くなることがあります。ご了承ください。
- ブラウザの戻るボタンで戻った場合、回答が正しく受け付けられない場合があります。その場合、再度アンケートを最初からやり直して下さい。

（調査NO. 14-406-0478）

NIKKEI-R

Q8A

あなたの家族の魚介類の消費量は、ここ15年間で変わりましたか。 （ひとつだけ）

- ○ かなり減った
- ○ 多少減った
- ○ 変わらない
- ○ 多少増えた
- ○ かなり増えた

Q8B

あなたの家族が食べる魚介類の種類は、ここ15年間で増えましたか。 （ひとつだけ）

- ○ はい
- ○ いいえ

- 回答内容により質問番号が飛ぶことがありますが、気にせずにご回答ください。
- 回線の混雑などが原因で反応が遅くなることがあります。ご了承ください。
- ブラウザの戻るボタンで戻った場合、回答が正しく受け付けられない場合があります。その場合、再度アンケートを最初からやり直して下さい。

Q9

ご家族の中で、泳げる方を教えてください。

（いくつでも）

- ☐ あなたご自身
- ☐ あなたの配偶者
- ☐ あなたの子供
- ☐ あなたのご両親
- ☐ あなたの兄弟・姉妹
- ☐ その他 （続柄をご入力ください： ＿＿＿＿＿＿ ）
- ☐ 全員泳げない

このページに入力したものを全てクリアする

一時保存をして中断

次へ

- 回答内容により質問番号が飛ぶことがありますが、気にせずにご回答ください。
- 回線の混雑などが原因で反応が遅くなることがあります。ご了承ください。
- ブラウザの戻るボタンで戻った場合、回答が正しく受け付けられない場合があります。その場合、再度アンケートを最初からやり直して下さい。

Q10

昨年の夏、（あなたを含む）あなたの世帯のどなたかが下表のレジャーを行なったことはありますか。

（それぞれひとつずつ）

	夏に毎週	夏に月1回	夏に2カ月に1回	夏に4回前後	夏に1回	していない
ビーチでの集まりやバーベキュー	○	○	○	○	○	○
シュノーケリング	○	○	○	○	○	○
ボディボード／サーフィン	○	○	○	○	○	○
キャンプ	○	○	○	○	○	○

▶ このページに入力したものを全てクリアする

▶ 一時保存をして中断

次へ ▶

- 回答内容により質問番号が飛ぶことがありますが、気にせずにご回答ください。
- 回線の混雑などが原因で反応が遅くなることがあります。ご了承ください。
- ブラウザの戻るボタンで戻った場合、回答が正しく受け付けられない場合があります。その場合、再度アンケートを最初からやり直して下さい。

沖縄の海洋環境に関する意識調査

start end

小 中 大 文字サイズ

Q11

昨年、（あなたを含む）あなたの世帯のどなたかが下表のレジャーを行ったことはありますか。

（それぞれひとつずつ）

	毎週	月に1回	2カ月に1回	年に4回前後	年1回	していない
カヤック	○	○	○	○	○	○
スキューバダイビング	○	○	○	○	○	○
釣り	○	○	○	○	○	○

▶ このページに入力したものを全てクリアする

▶ 一時保存をして中断

次へ ▶

- 回答内容により質問番号が飛ぶことがありますが、気にせずにご回答ください。
- 回線の混雑などが原因で反応が遅くなることがあります。ご了承ください。
- ブラウザの戻るボタンで戻った場合、回答が正しく受け付けられない場合があります。その場合、再度アンケートを最初からやり直して下さい。

(調査NO. 14-406-0478)

NIKKEI-R

Q12A

沖縄の海洋環境における下表の項目について、この10年間でどのように変化したと感じますか。

（それぞれひとつずつ）

	大幅に増加した	ある程度増加した	変わらない	やや減少した	大幅に減少した
生きたサンゴ礁の量	○	○	○	○	○
魚の量	○	○	○	○	○
魚の種類	○	○	○	○	○
海藻の生育	○	○	○	○	○
沈殿物	○	○	○	○	○
海水汚染	○	○	○	○	○

Q12B

沖縄の海洋環境における下表の項目について、この10年間でどのように変化したと感じますか。

（ひとつだけ）

	大幅に大きくなった	ある程度大きくなった	変わらない	やや小さくなった	大幅に小さくなった
魚の大きさ	○	○	○	○	○

このページに入力したものを全てクリアする

一時保存をして中断

次へ

- 回答内容により質問番号が飛ぶことがありますが、気にせずにご回答ください。
- 回線の混雑などが原因で反応が遅くなることがあります。ご了承ください。
- ブラウザの戻るボタンで戻った場合、回答が正しく受け付けられない場合があります。その場合、再度アンケートを最初からやり直して下さい。

沖縄の海洋環境に関する意識調査

start end

小 中 大
文字サイズ

Q13

沖縄の海洋環境に関して、ここ15年間で変化したと思う点があればご入力ください。

（半角カタカナは使用しないでください）

このページに入力したものを全てクリアする

一時保存をして中断

次へ

- 回答内容により質問番号が飛ぶことがありますが、気にせずにご回答ください。
- 回線の混雑などが原因で反応が遅くなることがあります。ご了承ください。
- ブラウザの戻るボタンで戻った場合、回答が正しく受け付けられない場合があります。その場合、再度アンケートを最初からやり直して下さい。

（調査NO. 14-406-0478）

NIKKEI-R

沖縄の海洋環境に関する意識調査

start end 文字サイズ 小 中 大

Q14

海岸線に面した住宅・宿泊施設・飲食店・道路の建設など、沖縄の沿岸開発は、どのような方針で進めるべきだと思いますか。

（ひとつだけ）

- ○ 規制せずに、すべての地域で沿岸開発を許可すべきである
- ○ 活動を規制し、特定の地域のみである程度の開発を許可すべきである
- ○ 沿岸地域での開発は禁止すべきである

Q15

Q14での回答の理由をお聞かせください。あてはまるものをすべてお選びください。

（いくつでも）

- □ 開発は海洋汚染をもたらすと思うから
- □ 開発により海の景観が損なわれると思うから
- □ 開発により海岸の民間利用が制限されると思うから
- □ その他 （具体的に：　　　　　　　　）

▶ このページに入力したものを全てクリアする

▶ 一時保存をして中断

次へ ▶

- 回答内容により質問番号が飛ぶことがありますが、気にせずにご回答ください。
- 回線の混雑などが原因で反応が遅くなることがあります。ご了承ください。
- ブラウザの戻るボタンで戻った場合、回答が正しく受け付けられない場合があります。その場合、再度アンケートを最初からやり直して下さい。

（調査NO. 14-406-0478）

NIKKEI-R

沖縄の海洋環境に関する意識調査

start end 文字サイズ 小 中 大

Q16

下記の行政サービスや政策について、重要だと思う順番にお答えください。

（上位から順番にお答えください）

海洋およびサンゴ礁の保全・保護
公共の安全・危機管理（法の執行）
教育
インフラ整備及び建設工事
観光業
社会福祉（高齢者・障害者・貧困者の福祉）
その他

第1位：
▽以下から選択
海洋およびサンゴ礁の保全・保護
公共の安全・危機管理（法の執行）
教育
インフラ整備及び建設工事
観光業
社会福祉（高齢者・障害者・貧困者の福祉）
その他

第2位：
▽以下から選択
海洋およびサンゴ礁の保全・保護
公共の安全・危機管理（法の執行）
教育
インフラ整備及び建設工事
観光業
社会福祉（高齢者・障害者・貧困者の福祉）
その他

第3位：
▽以下から選択
海洋およびサンゴ礁の保全・保護
公共の安全・危機管理（法の執行）
教育
インフラ整備及び建設工事
観光業
社会福祉（高齢者・障害者・貧困者の福祉）
その他

第4位：
▽以下から選択
海洋およびサンゴ礁の保全・保護
公共の安全・危機管理（法の執行）
教育
インフラ整備及び建設工事
観光業
社会福祉（高齢者・障害者・貧困者の福祉）
その他

第5位：
▽以下から選択
海洋およびサンゴ礁の保全・保護
公共の安全・危機管理（法の執行）
教育
インフラ整備及び建設工事
観光業
社会福祉（高齢者・障害者・貧困者の福祉）
その他

第6位：
▽以下から選択
海洋およびサンゴ礁の保全・保護
公共の安全・危機管理（法の執行）
教育
インフラ整備及び建設工事
観光業
社会福祉（高齢者・障害者・貧困者の福祉）
その他

第7位：
▽以下から選択
海洋およびサンゴ礁の保全・保護
公共の安全・危機管理（法の執行）
教育
インフラ整備及び建設工事
観光業
社会福祉（高齢者・障害者・貧困者の福祉）
その他

▶ このページに入力したものを全てクリアする

送信 ▶

- 回答内容により質問番号が飛ぶことがありますが、気にせずにご回答ください。
- 回線の混雑などが原因で反応が遅くなることがあります。ご了承ください。
- ブラウザの戻るボタンで戻った場合、回答が正しく受け付けられない場合があります。その場合、再度アンケートを最初からやり直して下さい。

NIKKEI-R
