# Supplementary material for: Impact of a local, coastal community based management regime when defining marine protected areas: Empirical results from a study in Okinawa, Japan
Source: PLoS One. 2019 Mar 8;14(3):e0213354. doi: 10.1371/journal.pone.0213354 (PMC6407762; doi:10.1371/journal.pone.0213354)
Supplement: S3 File — This pdf file provides an English translation of the centrally-managed MPA management regime survey document. In the translated version, we only include one choice scorecard. However, in the actual survey each respondent was provided with seven choice scorecards. (PDF) [file pone.0213354.s003.pdf]

EVALUATING PREFERENCES FOR MARINE ENVIRONMENT OF OKINAWA

This survey is designed to estimate the value of benefits provided by the marine resources in Okinawa. Participation is voluntary and will take approximately 30 minutes. You will not be asked to provide your name or address and your participation and answers to this survey are strictly confidential.

If you have any questions about this survey research or its results please contact:

Payal Shah, Ph.D., Research Scientist, payal.shah@oist.jp
Yoko Fujita, Director, Professor, yfujita@eve.u-ryukyu.ac.jp

If you have any questions about your rights as a participant in this study, please contact:

Eri Kanemoto, Research Safety Section
eri.kanemoto@oist.jp
Okinawa Institute of Science and Technology Graduate University
1919-1 Tancha, Onna-son, Kunigami-gun
Okinawa, Japan 904-0495

BACKGROUND INFORMATION:

The marine environment of Okinawa provides many services that are not bought and sold in the market such as free recreational opportunities, coastal protection, habitat for fish and other marine species. The environmental, economic, and social well-being of Okinawa depends on the marine environment's ability to provide a full range of these services. However, the Okinawan marine ecosystem faces a number of threats due to climate change and human activity. One solution to avoid these threats is to establish protected areas. The marine environment within protected areas enjoys a higher degree of protection relative to the surrounding areas. Such protection can allow the marine environment to recover from previous damage and mitigate possible future damage. The purpose of this survey is to find out whether people value key characteristics provided by the marine environment of Okinawa that do not have an observable market value.

SURVEY INSTRUCTIONS:

There are three sections in this survey. In the first section, we will ask you some questions to determine your level of involvement with the marine environment of Okinawa. In the second section, we will ask you to choose one among various available hypothetical policy scenarios for protecting the marine environment. For each choice, you can choose between three scenarios of possible benefits from marine resources. In the third section, we will ask you a series of short questions about yourself so that we can better understand what factors affect your choices for attribute combinations.

Please remember that your answers are strictly confidential.

PART ONE

The following information is important so that the researchers are able to verify that all groups in the Okinawa Region are represented. Remember that all responses are anonymous and strictly confidential.

1. Do you or anyone in your household have a job related to fishing?
   ❒ Yes ❒No
2. Do you have a friend who is a fisherman?
   ❒ Yes ❒No
3. Do you or anyone in your family have a job related to marine leisure?
   ❒ Yes ❒No
4. Do you have a friend who has a job related to marine leisure?
   ❒ Yes ❒No

5. Check **ALL** the following categories that describe you:
   1) Employed full time 2) Employed part time 3) Self-employed
   4) Student full time 5) Student part time 6) House wife/husband
   7) Retired 8) Unemployed 9) Other________________

6. What is your zipcode?

7. How many children under 18 live in your household? _______children

8. What is your gender? ❒Male ❒Female

9. What is your age? (exact age between 18 to 56; otherwise its 56 and above)

10. What category comes closest to your annual total household income? Check **ONE**.
    1) Less than 1,000,000 yen 2) Between 1,000,000 yen and 2,000,000 yen
    2) Between 2,000,000 yen and 5,000,000 yen 4) Between 5,000,000 yen and 7,000,000 yen
1) More than 7,000,000 yen 6) I don't know/I do not want to answer

11. Where were you born?
    ❒ Okinawa ❒ Mainland Japan ❒ Outside Japan

PART TWO

Next, imagine several protected areas are established by the central government to protect the marine ecosystem on the prefecture. We would like you to choose from a set of options that describe different levels of outcomes ten years from now for some key characteristics associated with the Okinawan marine environment. The outcomes are listed below.

Characteristic	Description	Current Conditions	Future Possible Levels in 10 years
Amount of captured fish	The average amount of fish catch available during a recreational fishing trip after 10 years.	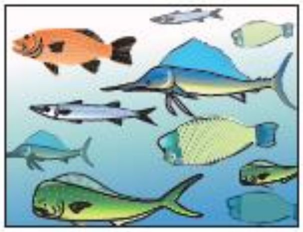	**With protected areas:**<br>1) 30% more fish catch<br>2) 15% more fish catch<br>3) Current conditions remain<br>**Without protected areas:**<br>1) 15% less fish catch
Coral Coverage and Marine Biodiversity	The extent and health of the coral reefs and the number of marine biodiversity found in the Okinawan waters after 10 years. Corals create habitats for numerous organisms and greater coral coverage is an indicator of healthy reefs and greater marine biodiversity.	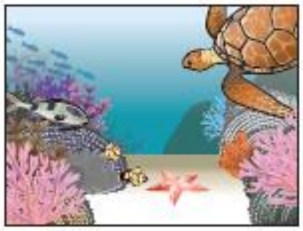	**With protected areas:**<br>1) 30% more coral coverage and biodiversity<br>2) 15% more coral coverage and biodiversity<br>3) Current conditions remain<br>**Without protected areas:**<br>1) 15% less coral coverage and biodiversity
Shoreline and Coastal Conditions	The extent of coastal development that include beach front construction of homes, hotels, restaurants and roads near or on coastal areas and the condition of the beach and shoreline after 10 years.	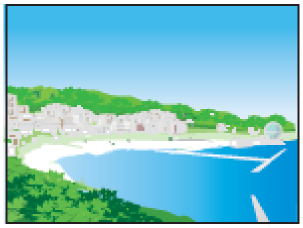	**With protected areas:**<br>1) 30% less development with more intact coastal shorelines<br>2) 15% less development and moderately intact coastal shoreline<br>3) Current conditions remain<br>**Without protected areas:**<br>1) 15% more development with degraded shoreline
Contribution/Donation	A monthly contribution will be collected from all Okinawan residents to support the management of these protected areas.	Zero	**With protected areas:**<br>1) 100 yen per month<br>2) 200 yen per month<br>3) 400 yen per month<br>4) 600 yen per month<br>5) 800 yen per month<br>6) 1000 yen per month<br>**Without protected areas:**<br>1) Zero

These monitoring and management activities will be funded by contributions from Okinawan residents. Based on the levels you see for each option, please choose the option that you would prefer.
Imagine you could choose one of the three options below. Option A and B represent possible future conditions after 10 years with protection for marine areas. Option C represents the possible future conditions (10 years from now) if no marine protected areas are established in Okinawa. Which would you choose?

	**Future Scenario A**<br>**Expected future after 10 years**	**Future Scenario B**<br>**Expected future after 10 years**	**Future Scenario C**<br>**Expected future after 10 years without any protective policy**
**Leisure Fish Catch**	**Current conditions remain**<br>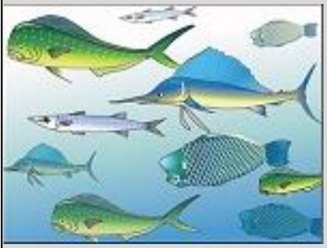	15% more<br>fish catch<br>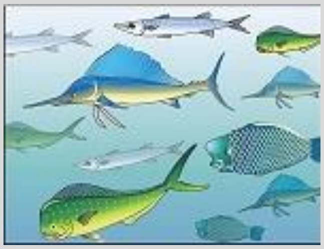	15% less<br>fish catch<br>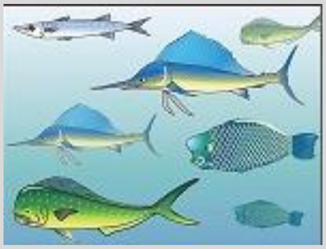
**Coral Coverage and Marine Biodiversity**	30% more<br>coral coverage and biodiversity<br>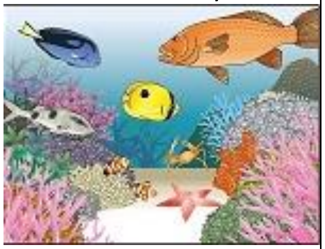	15% more<br>coral coverage and biodiversity<br>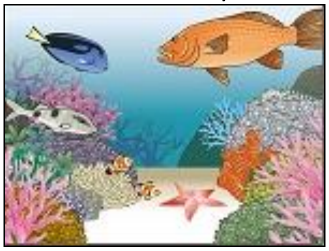	15% less<br>coral coverage and biodiversity<br>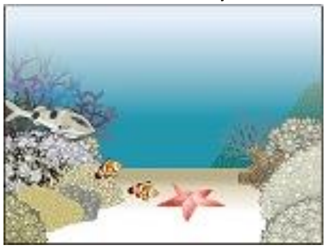
**Shoreline and Coastal Conditions**	Current conditions remain<br>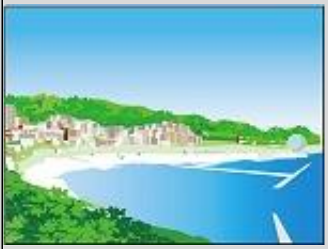	30% less<br>development with moderately intact shoreline<br>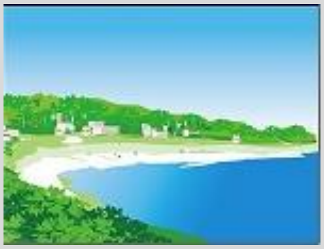	15% more<br>development with degraded shoreline<br>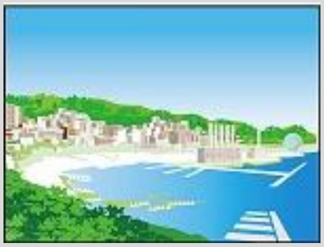
**Contribution**	600 yen per month	200 yen per month	Zero

I would choose ☐ Option A ☐ Option B ☐ Option C

PART THREE

1) My family eats fish/seafood at least once a week.
   Strongly Agree ____ Agree ____ Neutral ___ Disagree ____ Strongly Disagree ____

2) My family eats fish/seafood at least once a month.
   Strongly Agree ____ Agree ____ Neutral ___ Disagree ____ Strongly Disagree ____

3) I participate in recreational fishing activities at least once a week.
   Strongly Agree ____ Agree ____ Neutral ___ Disagree ____ Strongly Disagree ____

4) The fish/seafood we consume at home is caught by me or a family member.
   Strongly Agree ____ Agree ____ Neutral ___ Disagree ____ Strongly Disagree ____

5) The fish/seafood we consume at home is purchased from a local market.
   Strongly Agree ____ Agree ____ Neutral ___ Disagree ____ Strongly Disagree ____

6) In general, I and my family consume fish/seafood that comes from Okinawan reefs
   Strongly Agree ____ Agree ____ Neutral ___ Disagree ____ Strongly Disagree ____

7) In general, I and my family consume fish/seafood that is imported from mainland.
   Strongly Agree ____ Agree ____ Neutral ___ Disagree ____ Strongly Disagree ____

8) My family's fish seafood diet changed over the last 15 years such that we
   a. Eat much less fish___ Eat somewhat less fish___ No change___ Eat somewhat more fish ___Eat much more fish___
   b. We now eat different types (species) of fish.
      1) Yes 2) No

9) Please indicate who in your immediate family can swim (circle all that apply).
   a) Respondent
   b) Spouse
   c) Children
   d) Parents
   e) Siblings
   f) Other (please provide relationship)_____________________
   g) None

10) Last year, how often did anyone (including yourself) in your household participate in each of the following activities? Please check the relevant box for each activity.

Activity	Every Week during Summer	Once a month during Summer	Once in two months during Summer	±4 times during Summer	Once during Summer	Never
Beach Party/BBQ						
Snorkeling						
Body Boarding/Surfing						
Camping						

11) Last year, how often did anyone (including yourself) in your household participate in each of the following activities? Please check the relevant box for each activity.

Activity	Every Week	Once a month	Once in two months	±4 times a year	Once a year	Never
Kayaking						
Scuba Diving						
Fishing						

12)

a. In your opinion, how has the quality of the following components of the marine environment in Okinawa changed during the last 10 years?

	Increased Significantly	Increased somewhat	Remained stable	Decreased somewhat	Decreased significantly
Live coral abundance					
Fish abundance					
Fish species diversity					
Algae growth					
Sedimentation					
Water Pollution					

b. In your opinion, how has the quality of the following components of the marine environment in Okinawa changed during the last 10 years?

	Significantly Bigger	Somewhat bigger	Remained the same	Somewhat smaller	Significantly smaller
Fish Size					

13) Tell us what you think has changed in the past 15 years pertaining to the marine environment of Okinawa. ____

14) In your opinion, what should be the policy towards coastal development that include beach front construction of homes, hotels, restaurants and roads on Okinawan beaches?
    a) No restrictions- allow coastal development in all areas
    b) Restricted activity- allow some development in selected areas
    c) Not allow any development in coastal areas

15) Please tell us why you prefer the particular coastal development policy you chose in Q 15. Please choose all that apply.
    a) Development results in pollution of the sea
    b) Development obstructs the view of the ocean
    c) Development restricts public use of the beach
    d) Other reason. Please specify ________________________

16) How would you rank the importance of the following services:
    a) Marine and coral reef conservation and protection
    b) Public safety and security (law enforcement)
    c) Education
    d) Infrastructure and construction works
    e) Tourism
    f) Social welfare (welfare for elderly, handicapped and poor)
    g) Others
